# Comparative genomics of a vertically transmitted thiotrophic bacterial ectosymbiont and its close free‐living relative

**DOI:** 10.1111/1755-0998.13889

**Published:** 2023-11-27

**Authors:** Salvador Espada‐Hinojosa, Clarissa Karthäuser, Abhishek Srivastava, Lukas Schuster, Teresa Winter, André Luiz de Oliveira, Frederik Schulz, Matthias Horn, Stefan Sievert, Monika Bright

**Affiliations:** ^1^ Department of Functional and Evolutionary Ecology University of Vienna Vienna Austria; ^2^ Biology Department Woods Hole Oceanographic Institution Woods Hole Massachusetts USA; ^3^ Center for Microbiology and Environmental Systems Science University of Vienna Vienna Austria; ^4^ Present address: Deakin University Burwood Australia; ^5^ Present address: Max Planck Institute for Marine Microbiology Bremen Germany; ^6^ Present address: DOE Joint Genome Institute Berkeley California USA

**Keywords:** ectosymbiosis, low‐complexity metagenome, sulphur‐oxidizing bacteria, thiotrophy, *Zoothamnium niveum*

## Abstract

Thiotrophic symbioses between sulphur‐oxidizing bacteria and various unicellular and metazoan eukaryotes are widespread in reducing marine environments. The giant colonial ciliate *Zoothamnium niveum*, however, is the only host of thioautotrophic symbionts that has been cultivated along with its symbiont, the vertically transmitted ectosymbiont *Candidatus* Thiobius zoothamnicola (short Thiobius). Because theoretical predictions posit a smaller genome in vertically transmitted endosymbionts compared to free‐living relatives, we investigated whether this is true also for an ectosymbiont. We used metagenomics to recover the high‐quality draft genome of this bacterial symbiont. For comparison we have also sequenced a closely related free‐living cultured but not formally described strain Milos ODIII6 (short ODIII6). We then performed comparative genomics to assess the functional capabilities at gene, metabolic pathway and trait level. 16S rRNA gene trees and average amino acid identity confirmed the close phylogenetic relationship of both bacteria. Indeed, Thiobius has about a third smaller genome than its free‐living relative ODIII6, with reduced metabolic capabilities and fewer functional traits. The functional capabilities of Thiobius were a subset of those of the more versatile ODIII6, which possessed additional genes for oxygen, sulphur and hydrogen utilization and for the acquisition of phosphorus illustrating features that may be adaptive for the unstable environmental conditions at hydrothermal vents. In contrast, Thiobius possesses genes potentially enabling it to utilize lactate and acetate heterotrophically, compounds that may be provided as byproducts by the host. The present study illustrates the effect of strict host‐dependence of a bacterial ectosymbiont on genome evolution and host adaptation.

## 1 INTRODUCTION

**FIGURE 1:**
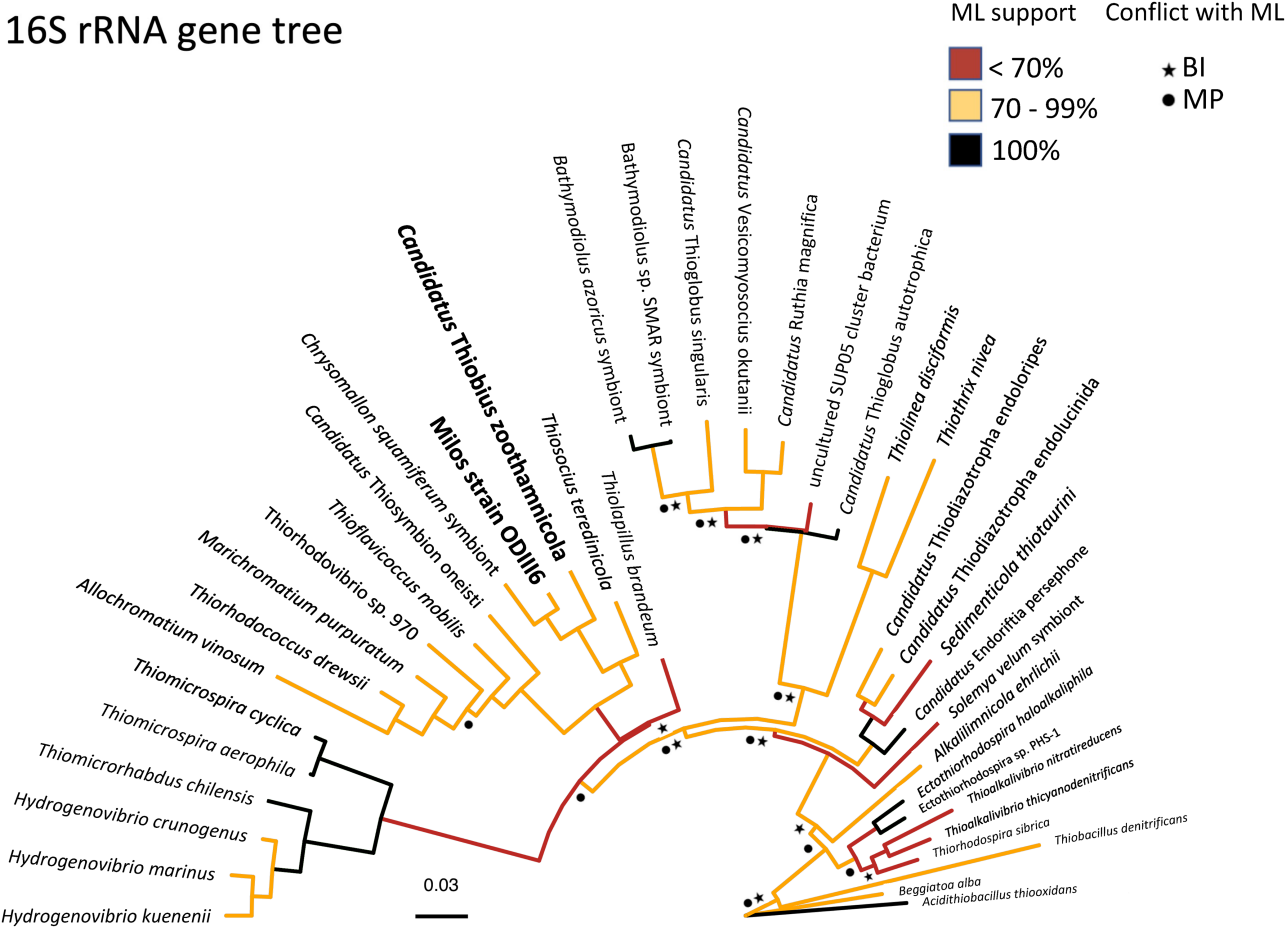
Maximum likelihood (ML) tree based on the 16S rRNA gene. Bootstrap support of the internal nodes is indicated with the colour of the outgoing branches. Topology conflicts with two other techniques Maximum parsimony (MP, ☻) and Bayesian inference (BI, ★) indicated with symbols in the affected internal nodes. The two microbes analysed in this study are highlighted in bold. The tree was generated with 306 sequences (Table S1), but only 40 organisms that have publicly released genomes are shown.

The beneficial associations between sulphur‐oxidizing bacteria (SOBs) and diverse protist and invertebrate hosts (Cavanaugh et al., 2006; Dubilier et al., 2008; Ott et al., 2004; Sogin et al., 2021; Stewart et al., 2005) span the entire range of ectosymbiotic mutualism, from the highly diverse microbiomes of alvinocarid shrimps and the low‐diversity microbiomes of alvinellid polychaetes at deep‐sea hydrothermal vents (Cambon‐Bonavita et al., 2021; Grzymski et al., 2008) to strictly single symbiont species such as those on stilbonematine nematodes from marine shallow‐water sediments (Ott et al., 1991; Paredes et al., 2021; Petersen et al., 2010; Polz et al., 1994), or on an amphipod in freshwater caves (Dattagupta et al., 2009). Among ciliates, monolayered coats of single ectosymbiotic SOB species are known from the karyorelictid *Kentrophoros* (Faure‐Fremiet, 1951; Fauré‐Fremiet, 1950; Fenchel & Finlay, 1989; Finlay & Fenchel, 1989; Raikov, 1971, 1974; Seah et al., 2019) and the peritrichs *Pseudovorticella* (Grimonprez et al., 2018; Laurent et al., 2009; Maurin et al., 2010) and *Zoothamnium* (Bauer‐Nebelsick et al., 1996a, 1996b; Hemprich & Ehrenberg, 1829, 1831; Rinke et al., 2006, 2009; Schuster & Bright, 2016). Some symbiotic SOBs form phylogenetic clusters with free‐living relatives (Figure 1; Dubilier et al., 2008). Here, we provide genomic information on two closely related SOBs with different lifestyles, that is, an obligate vertically transmitted ectosymbiont and a free‐living chemolithoautotrophic bacterium.

*Candidatus* Thiobius zoothamnicola of the Chromatiaceae (Chromatiales, Gammaproteobacteria) (Oren, 2017, short Thiobius) – originally introduced as *Candidatus* Thiobios zoothamnicoli (Rinke et al., 2006) – covers the surface of the giant colonial ciliate *Zoothamnium niveum* (short *Zoothamnium*) that can typically be found in shallow‐water environments from tropical to temperate waters with decaying organic material (Bauer‐Nebelsick et al., 1996a, 1996b, Rinke et al., 2006, 2009, reviewed in Bright et al., 2014). Thiobius is transmitted vertically (Bauer‐Nebelsick et al., 1996a, 1996b; Rinke et al., 2006) and has not been found free‐living despite extensive searches using general bacterial primers as well as a symbiont‐specific primer and direct Sanger sequencing (Monika Bright personal observation, 2022). To our best knowledge, the giant ciliate mutualism is the only thiotrophic symbiosis that has been cultivated in the laboratory over several host generations (Rinke et al., 2007). In contrast, the facultative endosymbiont *Thiosocius teredinicola* of the shipworm *Kuphus polithalamius* is the only thiotrophic symbiont that has been cultivated without its host (Distel et al., 2017).

The host *Zoothamnium niveum* grows fast and reproduces asexually developing specialized macrozooids that leave the colony as ectosymbionts‐covered propagules called swarmers to found new colonies upon settlement (Bauer‐Nebelsick et al., 1996a, 1996b). Thiobius thriving on the host surface fixes inorganic carbon using sulphide and provides organic carbon to the host (Volland et al., 2018). In return, benefits to the symbiont come through the host's peculiar contraction and expansion behaviour that ensures the supply of oxygen and sulphide (Rinke et al., 2007). The ciliate colonies can show unspecific overgrowth on the stalk, which connects them to the substrate surface (Bauer‐Nebelsick et al., 1996a).

Strain Milos ODIII6 (short ODIII6) is a cultured, but not yet formally described bacterium, that has been isolated from the shallow‐water hydrothermal vents in Paliochori Bay (Milos, Greece; Kuever et al., 2002; Sievert, 1999). ODIII6 is mesophilic (optimal growth at 34°C) and oxidizes reduced sulphur compounds under aerobic conditions.

Evolutionary theory predicts that the genome of obligate microbial symbionts, transmitted vertically from one to the next host generation, will be reduced in size compared to free‐living relatives and will contain a lower proportion of mobile elements than facultative symbionts (Newton & Bordenstein, 2011; Sachs et al., 2011). Being obligately host‐associated, these bacteria experience bottlenecks in population size during each transmission event leading to genome reduction (Bobay & Ochman, 2017; McCutcheon & Moran, 2012; Moran, 1996; Moran et al., 2009; Toft et al., 2009; Toft & Andersson, 2010; Wernegreen, 2015). Trait function compensation by the host or other symbionts can lead to further gene loss and reduced genome size (Ellers et al., 2012). Since obligate symbiotic bacteria are often isolated in the host as endosymbionts, they experience limited possibilities for horizontal gene transfer and access to foreign DNA. Some even lack the machinery for uptake and incorporation of DNA (Medina & Sachs, 2010). In contrast, obligate ectosymbionts have access to novel gene pools (Newton & Bordenstein, 2011). Nevertheless, some thermophilic ectosymbiotic archaea undergo genome reduction. This so‐called thermophilic streamlining was explained by having fewer and shorter genes in hot environments compared to archaea from other environments (Nicks & Rahn‐Lee, 2017). Much less studied are vertically transmitted bacterial ectosymbionts located on the surface of eukaryotic hosts. Mostly, these ectosymbiotic microbiomes are composed of complex microbial communities. Only a few cases with a single microbial partner are known and even fewer genomic studies are available (Fokin & Serra, 2022; Husnik et al., 2021).

Here, we show that the ectosymbiont Thiobius has a smaller genome, fewer genes, reduced GC content, and a smaller mobilome than the free‐living bacterium ODIII6, matching theoretical predictions. The analyses of metabolic capabilities revealed that the functional traits of Thiobius are largely a subset of the repertoire of ODIII6, which has a higher functional versatility to cope with broader environmental conditions as a free‐living bacterium from a highly unstable, fluctuating hydrothermal vent environment. In contrast, Thiobius shows a potential genetic capability to grow heterotrophically as an adaptation to its host.

## 2 MATERIALS AND METHODS

### 2.1 Specimen collection and cultivation

Three *Zoothamnium niveum* colonies were collected from attached submerged mangrove roots and wood at 1 m depth in 2015 at Twin Cays (Belize, 6°50′3″ N, 88°6′14″ W; strains G42, G43 and G44), and one colony from a sunken wood at 70 cm depth in 2014 at Guadeloupe (16°16′38″ N, 61°33′27″ W; strain G4). The ODIII6 isolate was originally obtained from the 10^−6^ dilution of a sediment sample collected at a sediment depth of 8–13 mm and at 2 m distance from the centre of a gaseous hydrothermal vent at a water depth of 8 m in Paliochori Bay (Milos Island, Greece, 36°40′23″ N, 24°31′13″ E) (Kuever et al., 2002; Sievert, 1999; Sievert et al., 1999). A culture frozen at −80°C in dimethylsulphoxide was reactivated in 2015 and used to obtain DNA for genome sequencing.

### 2.2 DNA preparation and sequencing

The lower parts of *Zoothamnium niveum* colonies are usually overgrown with diverse microbes (Bauer‐Nebelsick et al., 1996a; Rinke et al., 2006), and were therefore cut off to minimize contamination using Schreiber micro scissors (Fridingen, Germany, European Union). The upper parts, covered by a monolayer of Thiobius, were homogenized with Tris‐EDTA buffer by vortexing and DNA was extracted according to Zhou et al. (1996). The DNA extraction yielded up to 7 ng DNA per μL in ~30 μL of final volume for G43. Nextera XT (Illumina) DNA library preparation was used for multiplexing the *Zoothamnium* samples, and they were sequenced in a paired‐end mode with 125 nucleotides of read length using the Illumina HiSeqV4 platform at the Vienna Biocenter Core Facility (https://www.viennabiocenter.org/vbcf/). The low amount of DNA yielded by a single *Zoothamnium* ciliate colony precluded the use of long‐read sequencing technologies. The ODIII6 isolate was regrown from a frozen stock culture in 2015 and DNA was extracted with an UltraClean® microbial DNA isolation kit (MoBio laboratories). The DNA was sequenced with a MiSeq (Illumina) sequencer by a commercial provider, MR DNA (Shallowater, TX, https://www.mrdnalab.com/), using the 600 Cycles v3 Reagent Kit (Illumina). At MR DNA, the library of the sample was prepared with a Nextera DNA Sample Preparation Kit (Illumina).

### 2.3 Quality refinement, de novo assembly and binning

The sequenced reads of the four *Zoothamnium* samples were quality filtered with the *bbduk* command of the suite *bbmap* v35.92 (BBMap–Bushnell B.–sourceforge.net/projects/bbmap/) under a more stringent quality threshold of 25 (Phred units). The filtered paired‐end reads were assembled with *SPAdes* v3.7.1 in the metagenomic mode (Nurk et al., 2016). *MetaBAT* v0.26.3 (Kang et al., 2015) was then used for binning with default parameters. A nucleotide sequence homology *BLAST* search (Altschul et al., 1990) of the published 16S rRNA gene sequence of Thiobius (Rinke et al., 2006) identified the targeted bin. The genome of ODIII6 was de novo assembled by MR DNA using *NGEN* (DNAstar, https://www.dnastar.com). Completeness, heterogeneity and contamination of the ODIII6 and Thiobius assemblies were assessed with *CheckM* v1.0.5 (Parks et al., 2015). Presence of rRNAs and tRNAs for each amino acid was checked with *tRNAscan‐SE* (Lowe & Eddy, 1997). Average amino acid identity (AAI; Konstantinidis & Tiedje, 2005) was calculated with *CompareM* (https://github.com/dparks1134/CompareM). The assembly of ODIII6 with *SPAdes* was performed to exclude contamination, using the same set of reads as the MR DNA assembly. The alignment between both assemblies was generated and visualized with *Mauve* (Darling et al., 2010, version snapshot_2015‐02‐25), establishing the correspondence between the contigs. The fastg file containing paired‐end linkage information of the de Bruijn graph was visually inspected with *Bandage* v0.8.0 (Wick et al., 2015), allowing the assessment of physical connectivity between contigs of the bacterial chromosome.

### 2.4 Synteny analysis and localized gap filling

The four Thiobius draft genome assemblies were aligned with *Mauve* (Darling et al., 2010) and the ordering of the contigs was partly reconciled. A less stringent quality threshold of 20 (Phred units) was applied for the quality filtering and trimming of the G43 reads with *bbduk* resulting in the recovery of the full 16S rRNA gene.

### 2.5 Phylogenetic analyses

A total of 306 16S rRNA gene sequences were chosen following the taxa selection of Petersen et al. (2016) and Distel et al. (2017). 16S rRNA gene sequences were directly obtained from GenBank and RefSeq when available or extracted from publicly available genomes using *Metaxa* v2.2 (Bengtsson‐Palme et al., 2015). The alignment was done with *MAFFT* v7 (Katoh & Standley, 2013) and *TrimAl* v1.2 (Capella‐Gutierrez et al., 2009) was employed for trimming and filtering the alignments with default parameters. The obtained sequences were allocated to 292 putative species according to a 98.65% identity threshold (Kim et al., 2014; Table S1). Four species were excluded due to multiple divergent 16S rRNA gene copies (*Hydrogenovibrio halophilus*, *Lamprocystis purpurea*, *Thiofilum flexile* and *Thiothrix lacustris*). Phylogenetic analyses were performed using three methodological frameworks in order to assess the robustness of the tree inference: maximum parsimony (MP; Farris, 1970), maximum likelihood (ML; Felsenstein, 1981) and Bayesian inference (BI; Hastings, 1970). Packages *ape* v5.2 (Paradis et al., 2004) and *phangorn* v2.4.0 (Schliep, 2011) were used for MP and ML, and BI was computed with *MrBayes* v3.2.7a (Huelsenbeck & Ronquist, 2001). The substitution model choice followed a Bayesian information criterion (Schwarz, 1978) as implemented in *phangorn*. Bootstrap support (Felsenstein, 1985) in ML and MP trees was calculated with 100 trees in each, and in BI posterior probability was used as metric of support. To facilitate visualization, 16S rRNA gene trees were reduced by pruning most of the branches (Rodriguez‐Puente & Lazo‐Cortes, 2013) retaining only the 40 organisms with genomes available. By comparing the tree topologies, shared internal nodes and conflicting topologies were manually identified. We used ML tree as the base of the representation to show the support of the three approaches.

### 2.6 Functional inference of predicted genes and metabolic pathways

To predict and annotate genes the web version of *RASTtk* (https://rast.nmpdr.org, Brettin et al., 2015) was employed with default settings. Functional categories were assigned to the predicted genes by the use of *eggNOG mapper* version 2 (Huerta‐Cepas et al., 2017, 2019). Metabolic pathways were obtained using the predicted and annotated genes as an input by two independent methods, followed by a manual curation: (a) the online KEGG tool (www.kegg.jp, Kanehisa & Goto, 2000, Moriya et al., 2007) interrogating pathway complete modules; and (b) the *PathoLogic* module from *Pathway Tools* (v23.5, Karp et al., 2002, 2010) of *MetaCyc* (Caspi et al., 2008). To these two levels of granularity (the fine grain of the genes and the coarser grain of the metabolic pathways; Vogt, 2010) we added the overarching level of functional traits (De Oliveira et al., 2022), defined as microbial characteristics that can be observed and are linked to fitness (Green et al., 2008). Additionally, secretion, motility and defence potential capabilities encoded in the genomes were assessed with *MacSyFinder* (Abby et al., 2016). The potential presence of traits was determined by putting together constituent genes following *MetaCyc* and *KEGG* when available, or *MacSyFinder* for motility and interaction traits (Abby et al., 2016). Some traits were not detected by *MetaCyc* and *KEGG*, and therefore, were manually inferred based on scientific literature (Table S2). In the manual curation logical operators (AND, OR) were employed to combine the gene presence/absence evidence into traits, as outlined in Karaoz and Brodie (2022) (Table S2).

### 2.7 Orthology analysis

A total of 30 bacterial genomes, closest to Thiobius and ODIII6 based on the 16S rRNA gene tree phylogeny (Table S3), were selected excluding the genome of *Bathymodiolus* sp. SMAR symbiont due to poor quality of the assembly (52 scaffolds comprising 339 contigs with N_50_ as low as 10,280 nt). Protein sequences were clustered into orthologous groups using *OrthoFinder* v2.5.4 (Emms & Kelly, 2019).

## 3 RESULTS AND DISCUSSION

### 3.1 Close phylogenetic relationship of Thiobius and ODIII6

In order to confirm the previously reported close phylogenetic placement of Thiobius and ODIII6 (Distel et al., 2017; Lenk et al., 2011; Nunoura et al., 2014; Rinke et al., 2006, 2009; Schuster & Bright, 2016), 16S rRNA gene ML, MP and BI phylogenies were constructed. A pruned visualization of the resulting ML tree with indication of the conflicts between the three approaches is shown in Figure 1 (full ML tree in Figure S1). While some of the shallower internal nodes (closer to the tips) showed good support (e.g. bootstrap values greater than 70%) and agreement between the three phylogenetic approaches, many of the deeper internal nodes were poorly supported and showed topology conflicts. While ODIII6 falls in a clade containing the endosymbionts of the hydrothermal vent snails *Chrysomallon squamiferum* and *Alviniconcha* sp. Lau Basin, and free‐living bacteria, Thiobius can be assigned as sister taxon of this clade. The internal node connecting these clades obtained bootstrap supports of 70% (ML) and 63% (MP), while the posterior probability support of BI was 73%. The 16S rRNA gene sequences of Thiobius and ODIII6 were 95% identical (over 1396 aligned nucleotide positions). A relatively low average amino acid identity (AAI) of 67% was obtained for the whole genomes of Thiobius and ODIII6. Both 16S rRNA gene and AAI comparisons show that these two bacteria are related but ODIII6 is more closely related to both gastropod symbionts than to Thiobius (Distel et al., 2017, Lenk et al., 2011, Nunoura et al., 2014, Rinke et al., 2006, 2009, Schuster & Bright, 2016).

### 3.2 High‐quality draft genomes of Thiobius and ODIII6

**FIGURE 2:**
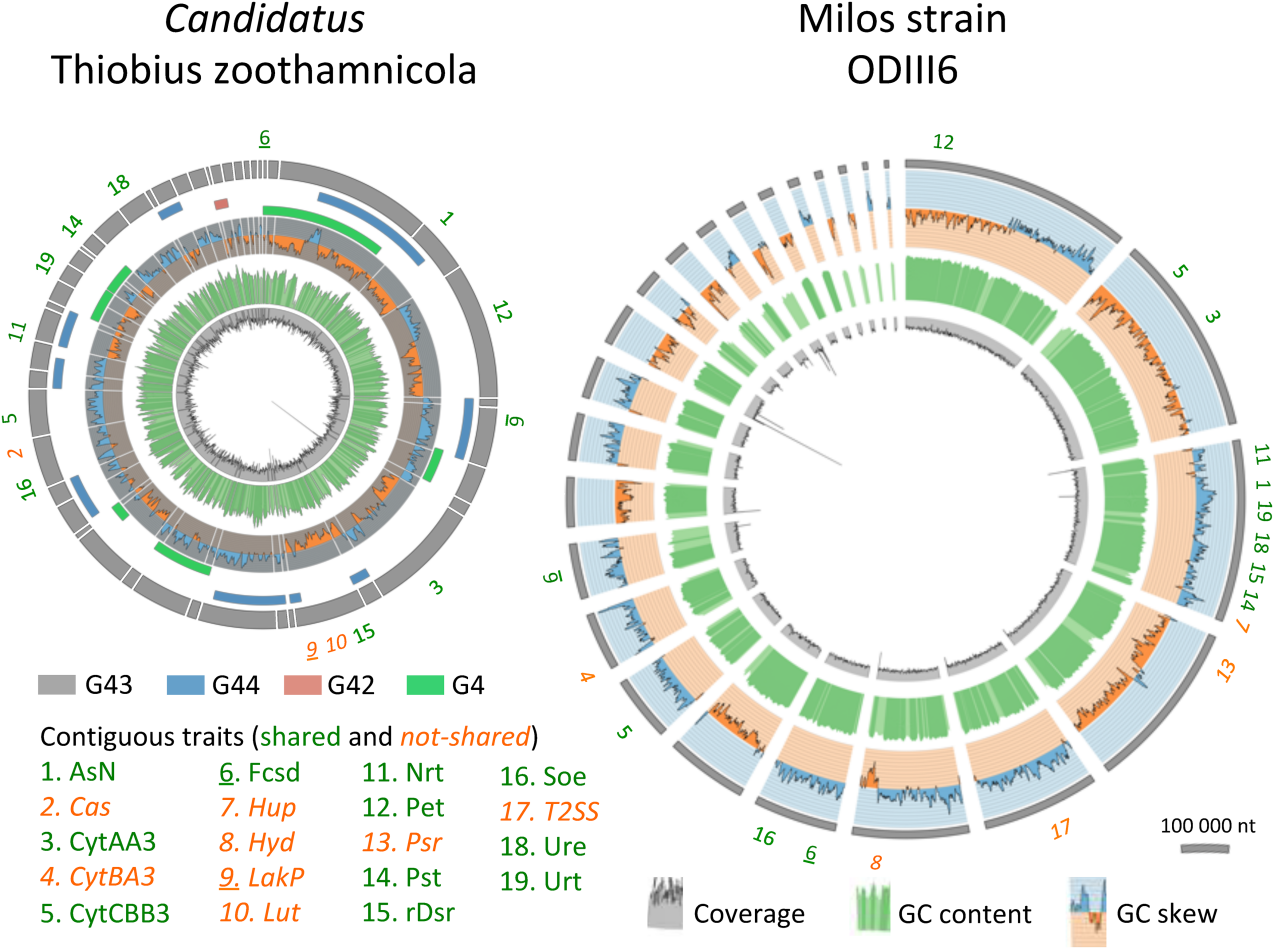
Representation of the draft genomes of Thiobius strain G43 (left) and ODIII6 (right). The most outer grey solid arcs represent the contigs. ODIII6 contigs are ordered by decreasing length. In Thiobius, information of three additional MAGs was employed to retrieve putative clusters of contiguity that are presented in decreasing length order. The bridging contigs are shown as solid arcs in colours, the rest of their contigs are omitted. GC skew typically reflects the attribution of the contig to the leading or to the lagging DNA strand. The correct ordering of the contigs has not been fully resolved, and this is reflected in the inconsistent GC skew pattern between neighbouring contigs. GC content and coverage are shown in the most inside rings. The location of relevant traits that show contiguity of their constitutive genes is also shown.

The best Thiobius metagenome assembled genome (MAG) in terms of assembly completeness and contiguity was obtained from a single ciliate colony collected in Belize (labelled G43, Figure S2; Tables 1 and S4). A total of 8.8 million of the initial 13.5 million read pairs passed a more stringent quality threshold filtering (phred 25). *SPAdes* assembled these reads into 44,538 contigs. *MetaBAT* binning grouped these contigs into four bins, one of them containing two partial matches at contig ends to the published 16S rRNA gene of Thiobius according to a BLAST search (Rinke et al., 2006; Figure S3). A less stringent quality threshold filtering (phred 20) led to a very similar *SPAdes* assembly that recovered the full 16S rRNA gene. The corresponding long contig containing the full 16S rRNA gene was used to replace three contigs from the stringent filtering assembly that covered the same span (Figure S4, Table S5). Overall, Thiobius G43 assembly resulted in 46 contigs with a total length of 2.38 Mb, a N_50_ value of 98,217, a coverage of 216× and a GC content of 49.4% (Figure 2). *CheckM* estimated its completeness as 96.0% and its contamination as 0.1%, and found no heterogeneity (Table 1), meeting the MIMAG standard for high‐quality MAGs (Bowers et al., 2017). Synteny analysis with *Mauve* between the four Thiobius MAGs led to the identification of 26 clusters of contiguity that were utilized in the final ordering of the contigs (Figures 2 and S2, Table S5).

**TABLE 1:** Statistics of the genomes assembled in this study.

	Thiobius str. BelizeG43	Thiobius str. BelizeG42	Thiobius str. BelizeG44	Thiobius str. GuadeloupeG4	ODIII6
Sequencing coverage	216×	296×	176×	131×	103×
Assembly size (bp)	2,381,364	2,379,254	2,311,386	2,369,374	3,528,654
GC content	49.4%	49.4%	49.6%	49.6%	61.9%
Number of contigs	46	47	57	46	25
N50 (bp)	98,217	98,217	66,776	73,149	245,984
Completeness	96.0%	96.0%	94.4%	96.0%	99.6%
Contamination	0.1%	0.1%	0.1%	0.4%	0.8%
Heterogeneity	0.0%	0.0%	0.0%	0.0%	0.0%
16S recovered	Yes	Partially	Partially	Yes	Yes
Number of extracted tRNAs	36	34	34	37	41
Amino acids with tRNA	20	19	19	20	20
Missing tRNA amino acid	–	Ile	Ile	–	–

ODIII6's genome MR DNA assembly consisted of 26 contigs, and had no paired‐end linkage associated information available (fastg file). Because *RASTtk* annotation revealed one contig with only phage related genes, we re‐assembled the genome using *SPAdes* and found through the paired‐end linkage information that the corresponding contig was circular and was thereby excluded (Figure S5). Accordingly, the final ODIII6 assembly resulted in 25 contigs, with a total of 3.53 Mb, a N_50_ value of 245,984, a coverage of 103×, and a GC content of 61.9%. *CheckM* estimated its completeness as 99.6% and its contamination as 0.8%, and found no heterogeneity either (Table 1).

A comparison between the high‐quality draft genomes of Thiobius and ODIII6 (both appropriate for general assessment of gene content, Chain et al., 2009), revealed a complete set of tRNAs for translation of all 20 amino acids in both bacteria. In agreement with theoretical predictions (Sachs et al., 2011), the genome of the vertically transmitted ectosymbiont Thiobius was 33% smaller than the one of the free‐living bacterium ODIII6, and showed a reduced relative GC content, consistent with the significant positive correlation between GC content and genome size in bacterial genomes (Almpanis et al., 2018). Whether the reduced genome size of the vertically transmitted ectosymbiont is due to any of the mechanisms that are known to affect vertically transmitted endosymbionts, such as those of thiotrophic vesicomyid clams (Kuwahara et al., 2007; Newton et al., 2007) and of marine flatworms *Paracatenula* (Jäckle et al., 2019), remains to be studied.

### 3.3 Thiobius encodes fewer genes, pathways and functional traits compared to ODIII6

To characterize the potential functional capabilities of Thiobius and ODIII6, their gene complements were predicted with *RASTtk* (Brettin et al., 2015). The annotation of Thiobius yielded 2486 predicted protein‐coding sequences, of which 1494 (60%) received a meaningful automatic functional prediction. In the manual curation process 21 hypothetical genes (18 non‐redundant) obtained a putative annotation. ODIII6 had 3452 predicted protein‐coding sequences including 2063 (60%) with meaningful functional predictions, and in the manual curation 21 hypothetical genes (19 non‐redundant) were functionally annotated. Thiobius contains about 28% less protein‐coding sequences than ODIII6. Accordingly, the lower number of protein‐coding sequences in Thiobius compared to ODIII6 is consistent with their genome sizes.

We manually selected 46 relevant functional traits involved in key metabolism and additional functions like storage or interactions, of these two SOBs. These traits can range from a single gene to the combination of several metabolic pathways (Tables 2 and S2). Less than half of the traits (19) showed a compact localization of their constitutive genes within contigs (Figure 2). *PathwayTools* inferred 203 *MetaCyc* metabolic pathways for Thiobius, while 39 complete *KEGG* modules were identified. The annotation of ODIII6 resulted in 233 *MetaCyc* pathways and 53 complete *KEGG* modules. Thiobius obtained 31 traits and ODIII6 yielded 39, of which 24 traits were shared (Table S6). Accordingly, Thiobius showed less pathways and relevant traits compared to ODIII6, particularly in those related with oxygen, phosphorus and sulphur.

**TABLE 2:** Selection of relevant functional traits.

Id	TraitGroup	TraitName	ShortTraitName	GeneTraitComposition	Thiobius	ODIII6
1	Carbon	Acetate transporter	ActP	+actP	Present	Absent
2	Carbon	Calvin‐Benson‐Bassham cycle	Cbb	+((+cbbS+cbbL),cbbL_formII) + pgk + gapA+tpiA+fbaA+fbp + tkt + rpe + prk + sir+rplA+PPi_PFK	Present	Present
3	Carbon	Glycogen biosynthesis	Glg	+glgC+glgA	Absent	Present
4	Carbon	Glyoxylate cycle	GlC	+mdh + CS+(acnA,acnB) + icl + aceB	Present	Absent
5	Carbon	Lactate transporter	LakP	+lakP+dctQ+dctM	Present	Absent
6	Carbon	Lactate utilization	Lut	+lutA+lutB+lutC	Present	Absent
7	Carbon	Polyhydroxyalkanoate synthesis	Pha	+pha	Present	Absent
8	Carbon	TCA cycle	TcaC	+CS+(acnA,acnB) + icd + ((+sucA+sucB+lpd),(+korA+korB)) + sucD+sucC+sdhA+sdhB+sdhC+sdhD+(fumA,fumC) + (mdh,mqo)	Present	Present
9	Carbon (and Nitrogen)	Cyanophycin synthesis	Cph	+cph	Absent	Present
10	Hydrogen	NiFe hydrogenase‐based hydrogen oxidation	Hup	+hupS+hupL	Absent	Present
11	Nitrogen	Ammonium transporter	AmtB	+amtB	Present	Present
12	Nitrogen	Assimilatory nitrate reduction	AsN	+narB+nirB+nirD	Present	Present
13	Nitrogen	Incomplete urea cycle	iUcy	+carA+carB+argF+argG+argH	Present	Present
14	Nitrogen	Nitrate transporter	Nrt	+nrtA+nrtB+nrtC	Present	Present
15	Nitrogen	Urea transporter	Urt	+urtA+urtB+urtC+urtD+urtE	Present	Present
16	Nitrogen	Urease mediated urea degradation	Ure	+ureA+ureB+ureC	Present	Present
17	Oxygen	Cytochrome aa3 based oxygen respiration	CytAA3	+coxA+coxB+coxC	Present	Present
18	Oxygen	Cytochrome ba3 based oxygen respiration	CytBA3	+cbaA+cbaB	Absent	Present
19	Oxygen	Cytochrome bc1 complex mediated electron transport	Pet	+petA+petB+petC	Present	Present
20	Oxygen	Cytochrome bd based oxygen respiration	CytBD	+ndhA_to_ndhN+shdA+sdhB+sdhC+cydA+cydB	Absent	Present
21	Oxygen	Cytochrome cbb3 based oxygen respiration	CytCBB3	+ccoN+ccoO+ccoP+ccoQ	Present	Present
22	Phosphorous	High‐affinity Na+/Pi symporter	Hnp	+hnp	Absent	Present
23	Phosphorous	High‐affinity phosphate transporter	Pst	+pstA+pstB+pstC+pstS	Present	Present
24	Phosphorous	Low‐affinity phosphate transporter	Pit	+pit	Absent	Present
25	Phosphorous	Polyphosphate usage	PHK and PHX	PHK, PHX	Present	Present
26	Sulphur	APR and SAT mediated sulphite oxidation	AprSat	+sat + aprB+aprA‐aprM	Present	Present
27	Sulphur	Reverse dissimilatory sulphate reductase mediated thiosulphate oxidation to sulphite	rDsr	+dsrA+dsrB+dsrC+dsrE+dsrF+dsrK+dsrH+tusA+rhd	Present	Present
28	Sulphur	Rhodanese mediated thiosulphate disproportionation	Tst	+tst	Absent	Present
29	Sulphur	Sulphate permease	SulP	+sulP	Absent	Present
30	Sulphur	Sulphate transporter	CysZ	+cysZ	Present	Present
31	Sulphur	Sulphydrogenase mediated sulphur reduction	Hyd	+hydA+hydB+hydC+hydD	Absent	Present
32	Sulphur	Polysulphide reductase	Psr	+psrA+psrB+psrC	Absent	Present
33	Sulphur	Sulphide dehydrogenase (flavocytochrome C) mediated sulphur oxidation	Fcsd	+fccA+fccB	Present	Present
34	Sulphur	Sulphide:quinone oxidoreductase mediated sulphur oxidation	Sqr	+sqr	Present	Present
35	Sulphur	Sulphite‐oxidizing enzyme mediated sulphite oxidation	Soe	+soeA+soeB+soeC	Present	Present
36	Sulphur	Sulphur globules proteins	Sgp	sgpCV1,sgpCV2,sgpA,sgpB,sgpC,sgpD	Present	Present
37	Sulphur	Sulphur oxygenase reductase mediated sulphur disproportionation	Sor	+sor	Absent	Present
38	Sulphur	Thiosulphate transporter	YeeE	+yeeE	Absent	Present
39	Sulphur	Truncated SOX mediated sulphur oxidation from thiosulphate to sulphate	tSox	+soxA+soxB+soxX+soxL	Present	Present
40	Interactions	CRISPR associated proteins	Cas	+cas1 + cas2 + ((cas3,cas7),cas9,cas10)‐cas4‐cas5‐cas6‐cas8‐cse1‐cse2‐csb1‐csb2‐cmr4‐cmr5‐cmr6‐csy1‐csy2‐csy3	Present	Absent
41	Interactions	Type I secretion system	T1SS	+lapB+lapC+tolC	Absent	Present
42	Interactions	Type II secretion system	T2SS	‐gspC+gspD+gspE+gspF+gspG‐gspH+gspI+gspJ+gspK‐gspL+gspM‐gspN	Present	Present
43	Interactions	Type V secretion system	T5SS	apeE,fhaC,yadA	Present	Present
44	Interactions	Type VI secretion system	T6SS	‐evpJ+tssA+tssB+tssC+tssD+tssE+tssF+tssG+tssH+tssI+tssJ+tssK+tssL+tssM	Present	Absent
45	Motility	Flagellum	Fla	+filF+filI+filN+filP+filQ+filR+flhB+flhA+flgB+flgC+filE	Absent	Present
46	Motility	Type IV pilus	T4P	+pilE+pilV+pilB+pilC+pilO+pilQ+pilM+pilN+pilP+pilT‐pilD	Present	Present

*Note*: ‘+’ indicates that the following gene is required, ‘‐’ indicates that the following gene is not required, ‘,’ indicates interchangeability; brackets are used for grouping; ‘ndhA_to_ndhN’ stands for 14 genes.

**FIGURE 3:**
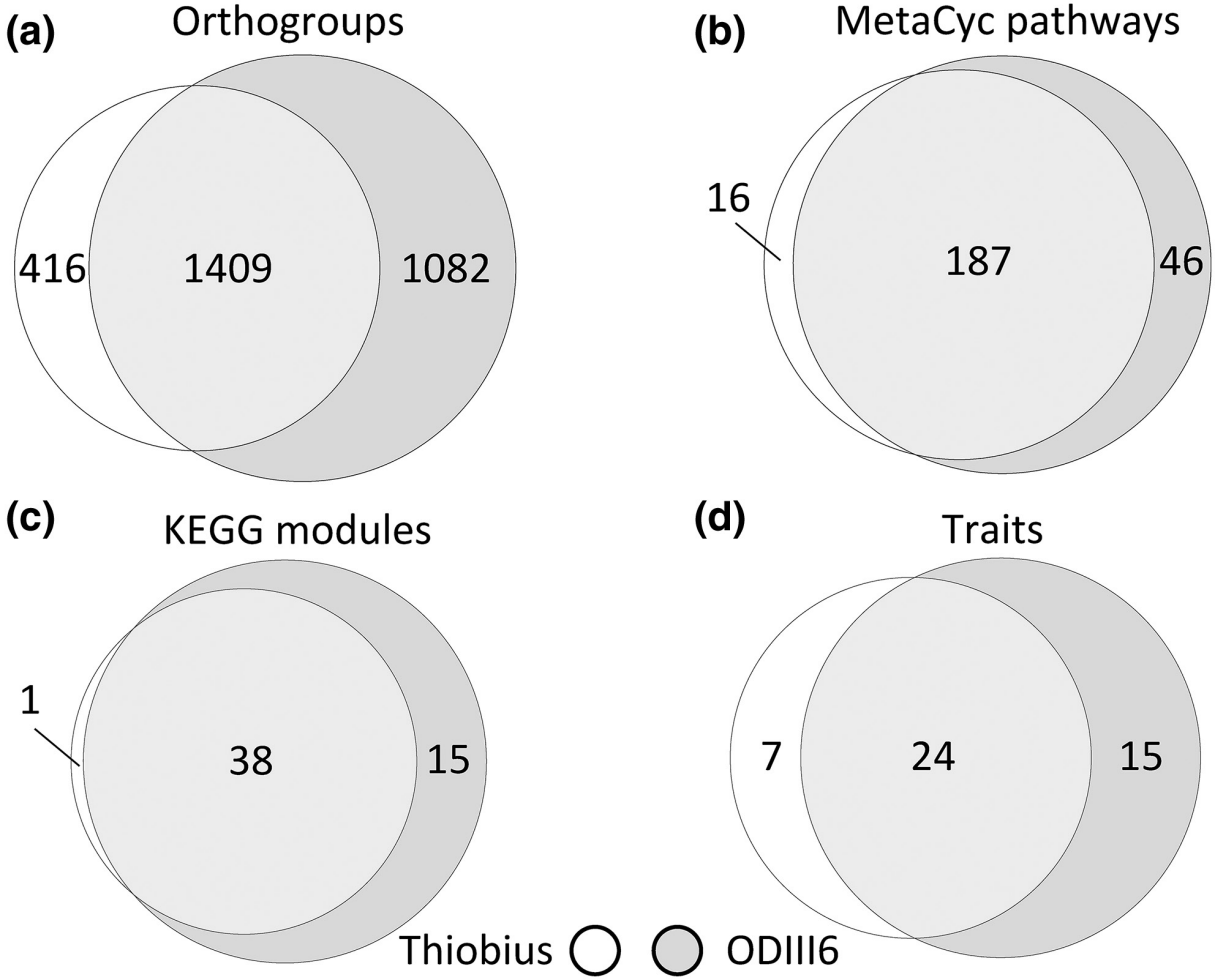
Euler diagrams of different levels of granularity showing shared and unique genetic potential capabilities of Thiobius and ODIII6. The finer granularity level is the genes from the orthogroups (a). Two metabolic pathway databases were employed: MetaCyc (b), and KEGG modules (c). The coarser granularity level are the traits (d).

In order to assess the functional commonalities between Thiobius and ODIII6 we performed an orthology analysis over a background set of 28 additional genomes of species included in the phylogenetic analysis (Table S3). Thiobius and ODIII6 shared 1409 orthogroups (Figure 3). A higher proportion of functional pathways were shared between the two organisms (Figure 3). Most of the genes of both organisms presented an orthologous one‐to‐one relationship (84% of genes in Thiobius and 90% of genes in ODIII6; Table S7). Overall, the relatively high percentage of shared orthogroups, pathways, modules and traits in Thiobius indicates that its functional capabilities are mainly a subset of those present in ODIII6 (Figure S6).

To further investigate the distribution of functional categories, *eggNOG* was applied to determine the attribution of COG categories (Tatusov et al., 2000). In general, the gene counts of the COG categories followed the genome sizes (Table S8), but there were some exceptions. ODIII6 and Thiobius COG categories yielded high count similarities in the categories ‘lipid metabolism’ and ‘secondary structure’. Nevertheless, this did not imply a fully identical sets of genes, for example, of the orthogroups involved in the ‘lipid metabolism’ category only a 52% were shared, indicating that different ‘non‐orthologous’ gene sets were recruited to perform same biological tasks in the two different bacteria. The least similar categories were ‘cell motility’ and ‘signal transduction’, for which the respective proportions were much lower in Thiobius in relation to its genome size (Table S8). Therefore, the COG functional categories composition reflects the free‐living lifestyle of ODIII6, with increased motility representation and a more complex gene regulation that match the requirements of a free‐living lifestyle.

### 3.4 Potential for a mixotrophic lifestyle as an adaptation to the ciliate host

Both Thiobius and ODIII6 show the genomic potential to fix inorganic carbon through the Calvin‐Benson‐Bassham cycle (Cbb) with the capability to form carboxysomes to concentrate ribulose‐1,5‐bisphosphate carboxylase oxygenase (RuBisCO; Badger & Bek, 2008), the key enzyme of the Calvin–Benson cycle responsible for CO_2_‐assimilation. According to the neighbouring genes the RuBisCO type in both Thiobius and ODIII6 is form IAc with a potential functional niche of low CO_2_ and low to high oxygen concentration (Badger & Bek, 2008). This is in line with previous studies in Thiobius, in which RuBisCO was histochemically detected, carboxysomes were identified by TEM (Bauer‐Nebelsick et al., 1996b) and a type IA RuBisCO large subunit sequence was retrieved (Rinke et al., 2009). In addition, carbon fixation in Thiobius was confirmed through tissue autoradiography and NanoSIMS (Volland et al., 2018). Further, the contraction and expansion behaviour of the host creates a continuously changing abiotic environment for Thiobius ranging from oxygen rich to sulphidic, anoxic conditions (Bright et al., 2014). ODIII6 shows two putative bicarbonate transporter‐encoding genes downstream of the carboxysome structural genes operon, consistent with previous reports on other chemoautotrophs (Axen et al., 2014; Scott et al., 2020). ODIII6 further possesses a gene for a beta class carbonic anhydrase not integrated in the carboxysome operon but elsewhere in the genome, which converts bicarbonate to CO_2_ for carbon fixation (Supuran & Capasso, 2017).

Organic carbon is stored differently in Thiobius compared to ODIII6. While Thiobius has the genetic potential for using polyhydroxyalkanoates (Pha), genes for glycogen (Glg) and cyanophycin synthesis (Cph; storing carbon and nitrogen) were found in ODIII6. In both organisms, the organic carbon is oxidized through the TCA cycle (TcaC), although. Thiobius encodes additionally genes for the glyoxylate cycle pathway (GlC; Cozzone & El‐Mansi, 2005). Putative genes encoding for transporters to import acetate (ActP) and lactate (LakP) along with genes encoding for all three components of L‐lactate dehydrogenase for lactate utilization (Lut) were also present. Both acetate and lactate could potentially be metabolized by the glyoxylate cycle (El‐Mansi et al., 1986; Serafini et al., 2019). While in many organisms (including *Bacillus subtilis*) *lutABC* belongs to the same operon with a lactate permease (Chai et al., 2009), Thiobius possesses a different DctP–TRAP‐like transporter (LakP), similar to the one described for *Thermus thermophilus* (Fischer et al., 2010). The presence of these transporters indicates a potential for heterotrophic metabolism in Thiobius. As the ciliate host may potentially be able to switch to an anaerobic metabolism under sulphidic conditions and produce lactate and acetate, similarly to the rumen ciliate *Entodinium caudatum* (Park et al., 2021), it is tempting to speculate that these fermentation products may then be released from the host and taken up by the symbiont, but these anaerobic processes have yet to be studied in this mutualism. No equivalent capabilities were found in the genome of ODIII6 in line with its obligate autotrophic metabolism.

### 3.5 Oxygen is the only electron acceptor in both bacteria, but ODIII6 is more versatile than Thiobius

According to the genomic potential of their draft genomes, oxygen is the only electron acceptor that Thiobius and ODIII6 can utilize. Genes for nitrate respiration, known in many free‐living and symbiotic thiotrophic bacteria living at oxic–anoxic interfaces (De Oliveira et al., 2022; Flood et al., 2015; König et al., 2016; Nunoura et al., 2014; Paredes et al., 2021), were not found. This indicates that both bacteria strongly rely on oxygen both for respiration and for the oxidation of reduced sulphur species to generate energy. Indeed, both genomes encode for a cytochrome bc_1_ electron transport complex (Pet), and two cytochrome terminal oxidases, that is, cbb_3_ (CytCBB3), which was shown to have a high affinity for oxygen in *Bradyrhizobium japonicum* (Pitcher & Watmough, 2004), and aa_3_ (CytAA3), which belongs to the Class A of oxidases with lower apparent oxygen affinities (Han et al., 2011). This indicates the ability to utilize oxygen at various concentrations. Interestingly, the genome of ODIII6 contains two copies of the genes encoding cbb_3_, which is unusual and could be an additional adaptation to optimize oxygen utilization under varying concentrations, as was shown for *Pseudomonas aeruginosa* (Comolli & Donohue, 2004). Further, ODIII6 possesses two more high‐affinity terminal oxidases, cytochrome bd (CytBD)‐ and ba_3_ (CytBA3)‐encoding genes, which points to a higher versatility under a broader range of oxygen regimes than Thiobius. In contrast, Thiobius lives on a ciliate host that can position itself in microhabitats with optimal oxygen concentrations, which potentially renders the ability to express multiple terminal oxidases unnecessary.

### 3.6 Both organisms use the oxidation of reduced sulphur compounds to generate energy

The energy fuelling processes for carbon fixation in Thiobius and ODIII6 comes from the oxidation of reduced sulphur species using oxygen as terminal electron acceptor. Sulphide oxidation to elemental sulphur occurs in both bacteria through two possible pathways, sulphide dehydrogenase (Fcsd; flavocytochrome C, Sorokin et al., 1998), and sulphide:quinone oxidoreductase (Sqr). ODIII6 has a type VI Sqr and Thiobius has type I and type VI Sqr (Dahl, 2017). The elemental sulphur formed by sulphide oxidation is stored in sulphur globules that are enveloped by proteins (Dahl, 2017) identified in both genomes (Sgp). Indeed, transmission electron micrographs (Bauer‐Nebelsick et al., 1996b) and Raman microspectrometry previously revealed membrane bound elemental sulphur vesicles in Thiobius (Maurin et al., 2010) used to store sulphur under sulphidic conditions and oxidize it further during oxic conditions (Volland et al., 2018). In ODIII6 cultures, the formation of sulphur globules attached to the cell could also be observed if thiosulphate was provided (Stefan Sievert personal observation, 2022).

**FIGURE 4:**
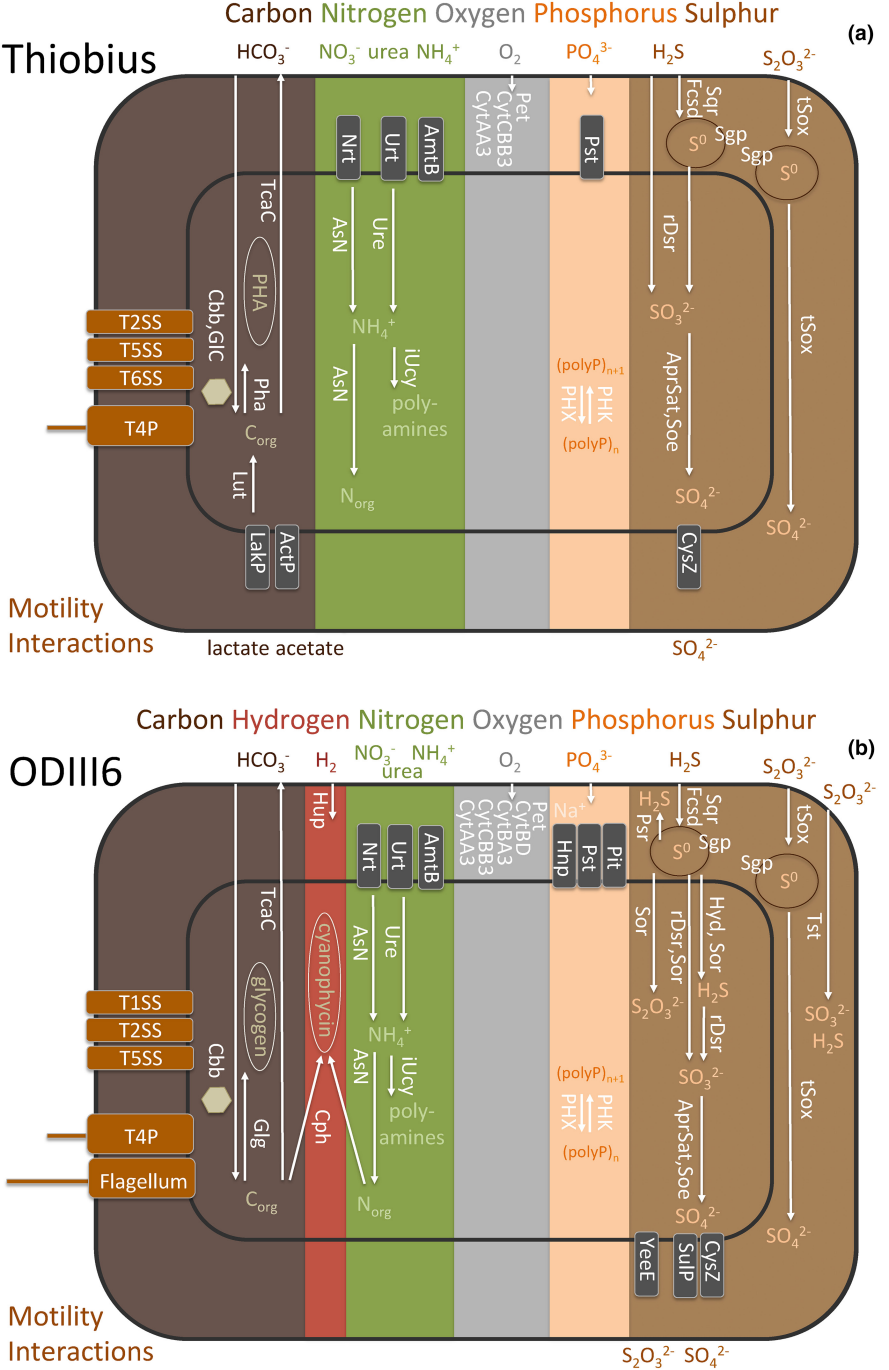
Major metabolic capabilities found in the draft genomes of (a) *Candidatus* Thiobius zoothamnicola strain BelizeG43, and (b) strain Milos ODIII6. Chosen relevant potential functional capabilities are shown (for simplicity not all transporters are depicted in the figure). Predicted and functionally annotated genes are grouped in metabolic pathways. Functional traits are features such as single metabolic pathways or composites of them that affect the organism fitness. Structural features such as transporters or secretion systems, and storage capabilities are also considered as traits. Trait labels are generally in white fonts and in vertical orientation, except for the motility and interactions on the left. Compound labels are horizontally oriented. Storage compartments are indicated with ellipses. ActP, acetate transporter; AmtB, ammonium transporter; AprSat, sulphate adenylyltransferase and adenylylsulphate reductase sulphite oxidation; AsN, assimilatory nitrate reduction; Cbb, Calvin‐Benson‐Basham cycle; Cph, cyanophycin biosynthesis; CysZ, sulphate transporter; CytAA3, cytochrome aa_3_ based oxygen respiration; CytBA3, cytochrome ba_3_ based oxygen respiration; CytBD, cytochrome bd based oxygen respiration; CytCBB3, cytochrome cbb_3_ based oxygen respiration; Fcsd, flavocytochrome c sulphide dehydrogenase sulphide oxidation; GlC, glyoxylate cycle; Glg, glycogen biosynthesis; Hnp, high‐affinity sodium‐phosphate symporter; Hup, putative hydrogen oxidation; Hyd, sulphydrogenase elemental sulphur oxidation; iUcy, incomplete urea cycle lacking last step arginase gene; LakP, lactate transporter; Lut, lactate utilization; Nrt, nitrate transporter; Pet, cytochrome bc_1_ complex mediated electron transport chain; Pha; polyhydroxyalkanoate synthesis; PHK, polyphosphate kinase; PHX, exopolyphosphatase; Pit, low‐affinity phosphate transporter; Psr, polysulphide reductase; Pst, high‐affinity phosphate transporter; rDsr, reverse dissimilatory sulphate reductase mediated sulphur oxidation; Sgp, sulphur globule proteins; Soe, sulphite‐oxidation enzyme sulphite oxidation; Sor, Sulphur oxygenase reductase mediated sulphur oxidation; Sqr, sulphide: quinone oxydoreductase sulphide oxidation; SulP, sulphate permease; T1SS, type I secretion system; T2SS, type II secretion system; T5SS, type V secretion system; T6SS, type VI secretion system; T4P, type IV pilus; TcaC, TCA cycle; tSox, truncated Sox mediated sulphur oxidation; Tst, thiosulphate disproportionation; Ure, Urease mediated urea degradation; Urt, urea transporter; YeeE, thiosulphate transporter. The hexagon represents a carboxysome.

Thiosulphate is potentially oxidized in both organisms to sulphate and elemental sulphur through the truncated Sox pathway (tSox, Welte et al., 2009, Dahl, 2020), with the possible involvement of *SoxL* (Weissgerber et al., 2011). For both organisms, elemental sulphur is oxidized to sulphite in the cytoplasm by the reverse dissimilatory sulphate reductase pathway (rDsr; Dahl, 2015, Gregersen et al., 2011, Hensen et al., 2006), and sulphite is oxidized to sulphate either by the adenylylsulphate reductase and the sulphate adenylyltransferase (AprSat), or by the sulphite‐oxidizing enzyme (Soe; Dahl, 2017). Gene sequences for the alpha and beta subunits of the reverse‐type dissimilatory sulphite reductase (*dsrAB*) and for the alpha subunit of the adenylylsulphate reductase (*aprA*) were previously reported in Thiobius (Rinke et al., 2009). Finally, the putative sulphate transporter CysZ exports the sulphate to the periplasm (Figure 4a; Hryniewicz et al., 1990).

In contrast to Thiobius, ODIII6 has additional sulphur‐metabolizing capabilities: the periplasmic disproportionation of thiosulphate by a rhodanese‐like sulphurtransferase to sulphide and sulphite (Tst; Deckert et al., 1998); elemental sulphur reduction to sulphide in the periplasm by a putative polysulphide reductase (Psr; De Oliveira et al., 2022), that might also be involved in oxidation as suggested for *Allochromatium vinosum* (Weissgerber et al., 2013); cytoplasmic disproportionation of elemental sulphur to thiosulphate, sulphite and sulphide by the sulphur oxygenase reductase (Sor; Janosch et al., 2015), as suggested for *Thioalkalivibrio paradoxus* (Rühl et al., 2017); reduction of elemental sulphur to sulphide coupled to the oxidation of hydrogen by the sulphydrogenase (Hyd; Ng et al., 2000); and sulphate exportation to the periplasm by the sulphate permease SulP (Figure 4b; Aguilar‐Barajas et al., 2011). A thiosulphate transporter gene (YeeE; Tanaka et al., 2020) is also found in the genome of ODIII6.

Although many sulphur oxidation traits are shared, we observe a higher versatility in ODIII6 than in Thiobius. In addition, the presence of [NiFe] hygrogenase Hup genes (Vignais et al., 2001) in ODIII6 may indicate the potential of hydrogen oxidation. However, it was recently found in *Candidatus* Endoriftia persephone that *hup* genes present in its genome were not involved in hydrogen oxidation but may instead facilitate intracellular redox homeostasis (Mitchell et al., 2019). The two subunits L and S in ODIII6 show 75% and 71% amino acid identity with their *Candidatus* Endoriftia persephone orthologues.

### 3.7 Similarities in nitrogen and phosphorous metabolism

Both organisms show similar potential capabilities in nitrogen metabolism. Nitrate is imported into the cytoplasm through the nitrate transporter Nrt (Maeda et al., 2019), and is reduced to ammonium in the first step of assimilatory nitrate reduction, and from there assimilated as organic nitrogen in form of biomolecules (AsN; Takai, 2019). In addition, the ammonium can be imported into the cytoplasm by the ammonium transporter AmtB (Wang et al., 2012) further fuelling assimilation. Urea is acquired by the urea transporter Urt and oxidized to ammonium by the urease (Ure; Bossé et al., 2001). An incomplete urea cycle lacking arginase is also present (iUcy; De Oliveira et al., 2022). Overall, urea as well as ammonium are well‐known nitrogen waste products of ciliates (Caron & Goldman, 1990), and may serve as byproducts the host provides to the symbiont. Urea was shown to be a source of incorporated nitrogen for bacteria in intertidal sediments (Veuger & Middelburg, 2007). Whether urea is utilized by ODIII6 in its natural environment remains to be studied.

Phosphorus is a limiting nutrient in many marine environments, present in its inorganic dissolved fraction generally as orthophosphate (Paytan & McLaughlin, 2007). Both organisms encode the genes for a high‐affinity phosphate transporter (Pst), and additionally ODIII6 possesses genes for two other transporters: a high‐affinity Na^+^/P_i_ symporter (Hnp) and a low‐affinity phosphate transporter (Pit). Once phosphate is incorporated into the cells, both organisms also show the potential for polymerizing it into polyphosphate and hydrolysing back to inorganic phosphates (PHKandPHX), using it as energy storage (Achbergerová & Nahálka, 2011).

### 3.8 Interaction and motility in both organisms

Both Thiobius and ODIII6 have a repertoire of genes to interact with other organisms and with the environment. They share genes for the type II (T2SS) and type V (T5SS) secretion systems. Thiobius additionally has genes for the type VI secretion system (T6SS; Kapitein & Mogk, 2013) known to function in host interaction (Hachani et al., 2016) and CRISPR‐Cas proteins (Cas), while ODIII6 has genes for the type I secretion system (T1SS). It remains to be studied how both bacteria use these traits in their natural environment and whether they may help Thiobius to interact with its host.

Both organisms further possess genes for type IV pilus potentially involved in twitching motility (T4P; Ayers et al., 2010), and additionally ODIII6 genome contains several loci for the biosynthetic genes encoding for a flagellum (Fla). Indeed, ODIII6 was observed to be motile in cultures, however, loses its motility after a longer period of cultivation (Sievert pers. obs.). This suggests that the flagellum may no longer be expressed in ODIII6 if constant‚ favourable conditions render motility unnecessary. ODIII6 additionally shows genes for photolyase DNA protection against UV radiation, and repair (Sancar et al., 1987).

### 3.9 Mobile genetic elements are less abundant in Thiobius than in ODIII6

Bacteria can experience horizontal gene transfer through mobile genetic elements such as phages, plasmids, transposons and insertion sequences, also referred to as the mobilome (Frost et al., 2005). Free‐living bacteria like ODIII6 are more likely to be exposed to novel gene pools than symbionts (Newton & Bordenstein, 2011). In addition, vertically transmitted symbionts, such as Thiobius experience population bottlenecks as each swarmer is covered with relatively few symbionts that grow to cover the new colony (Bauer‐Nebelsick et al., 1996a, 1996b). Accordingly, we hypothesized a smaller mobilome in the obligate symbiont Thiobius than in the free‐living ODIII6. Indeed, *RASTtk* annotation of Thiobius revealed three genes attributed to phages and 18 genes attributed to other mobile genetic elements, while ODIII6 has two genes attributed to phages and 58 genes attributed to other mobile genetic elements (Table S9). Roughly 60% of these mobile elements were located at the extremes of the contigs, consistent with their disruptive effect on the assembly processes. These results point to a smaller mobilome in the host‐associated Thiobius than in the free‐living ODIII6.

## 4 CONCLUSIONS

The phylogenetic relationship of the ectosymbiont Thiobius and the strain Milos ODIII6 is confirmed through 16S rRNA phylogeny and average amino acid identity and serves as baseline to compare the genomes of these two bacteria with very different lifestyles. In agreement with theoretical predictions, but hardly studied in ectosymbiotic bacteria, Thiobius' genome is smaller than that of its free‐living relative ODIII6. The characterization at the levels of genes, metabolic pathways and traits reveals that Thiobius and ODIII6 share a large proportion of their genetic repertoire and metabolic capabilities. The lower number of lineage‐specific metabolic pathways and relevant traits in Thiobius compared to ODIII6 may point to a more stable environment provided by the host, requiring less versatility. This may have led to a loss of genetic potential in Thiobius and/or gain in ODIII6. In comparison with Thiobius, ODIII6 shows a larger functional repertoire, in particular for its energy metabolism regarding the utilization of sulphur, oxygen and hydrogen, consistent with the requirements for a free‐living bacterium to live under the fluctuating conditions of the hydrothermal vent environment. Thiobius, however, shows potential for heterotrophic metabolism, which may be fuelled by byproducts from the host and thus might represent a remarkable adaptation to the life style of its protist host. In contrast to reduced genomes of vertically transmitted, thiotrophic endosymbionts like those of vesicomyid clams or catenulid plathylhelmints that experience no microbial competition and little potential for horizontal gene transfer inside host organs and cells, Thiobius, as an ectosymbiont, faces potential competitive interactions and viral attacks similar to free‐living bacteria such as ODIII6, which is reflected in its capacity to interact with the environment. In the future, transcriptome evidence and other omics analyses in conjunction with physiological experiments can elaborate on the intricacy of these functional capabilities.

## AUTHOR CONTRIBUTIONS

S.E.‐H., C.K., S.M.S. and M.B. designed the research. S.E.‐H. performed the DNA extraction and preparation, and synteny analyses (together with T.W.) for Thiobius, the final versions of genome annotation for Thiobius and ODIII6, the phylogenies, the orthology analyses (together with A.L.de.O.) and reassembled ODIII6 with SPAdes. F.S. and L.S. performed the assembly and binning of Thiobius. M.H. provided valuable input for DNA extraction, assembly, binning and annotation of Thiobius, S.E.‐H., A.S. and M.B. manually curated the functional inference results. S.M.S. isolated ODIII6 from the environment, helped C.K. to grow ODIII6 for DNA extraction, and provided input on gene annotation and pathway inference. C.K. extracted DNA of ODIII6, carried out an initial comparison of the two genomes and their genome contents using PathwayTools and KEGG modules, and provided a summary as part of a MSc thesis. S.E.‐H. wrote the initial draft of the manuscript and C.K., S.M.S. and M.B contributed considerably to the writing. All authors commented and approved the final version of the manuscript.

## CONFLICT OF INTEREST STATEMENT

The authors declare no conflict of interest.

## Supporting information


Figure S1.

Figure S2.

Figure S3.

Figure S4.

Figure S5.

Figure S6.

Table S1.
Table S2.
Table S3.
Table S4.
Table S5.
Table S6.
Table S7.
Table S8.
Table S9.

## Data Availability

Thiobius and ODIII6 Whole Genome Shotgun projects have been deposited at DDBJ/ENA/GenBank under the BioProjects PRJNA906600 (Thiobius) and PRJNA910104 (ODIII6), with the following accessions (G43) JAPQLC000000000; (G42) JAPUCE000000000; (G44) JAPUCF000000000; (G4) JAPUCG000000000 and (ODIII6) JAPTHR000000000. The Illumina reads are also accessible in the SRA associated entries. All other data and code files are available from the Dryad repository (https://doi.org/10.5061/dryad.wh70rxwrq).
