# Supplementary figures and images for: Comparative genomics of a vertically transmitted thiotrophic bacterial ectosymbiont and its close free‐living relative
Source: Mol Ecol Resour. 2023 Nov 27;24(1):e13889. doi: 10.1111/1755-0998.13889 (PMC10952691; doi:10.1111/1755-0998.13889)

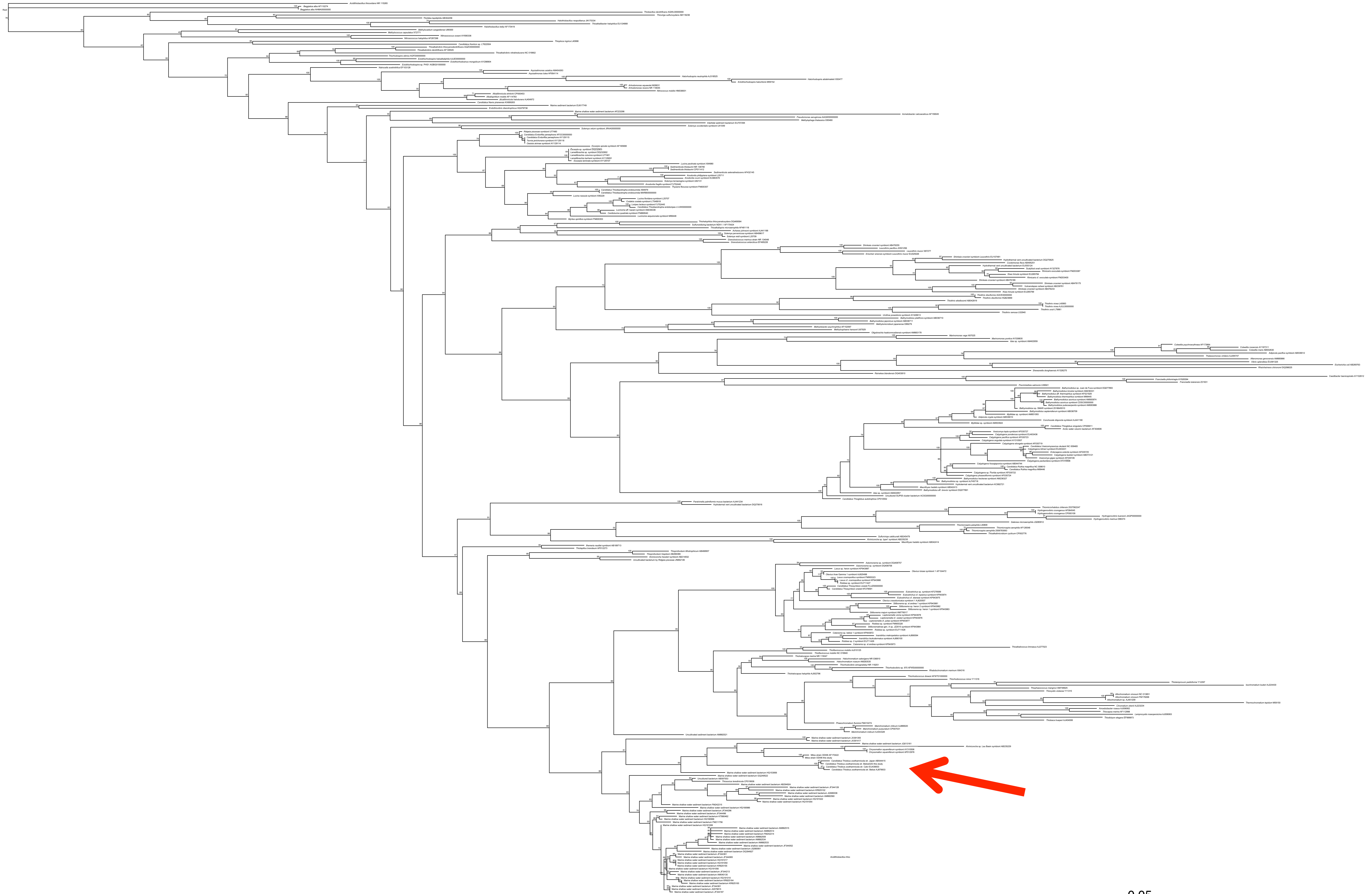

Supplement: Supplementary file 1 — Figure S1. [file MEN-24-0-s003.pdf]

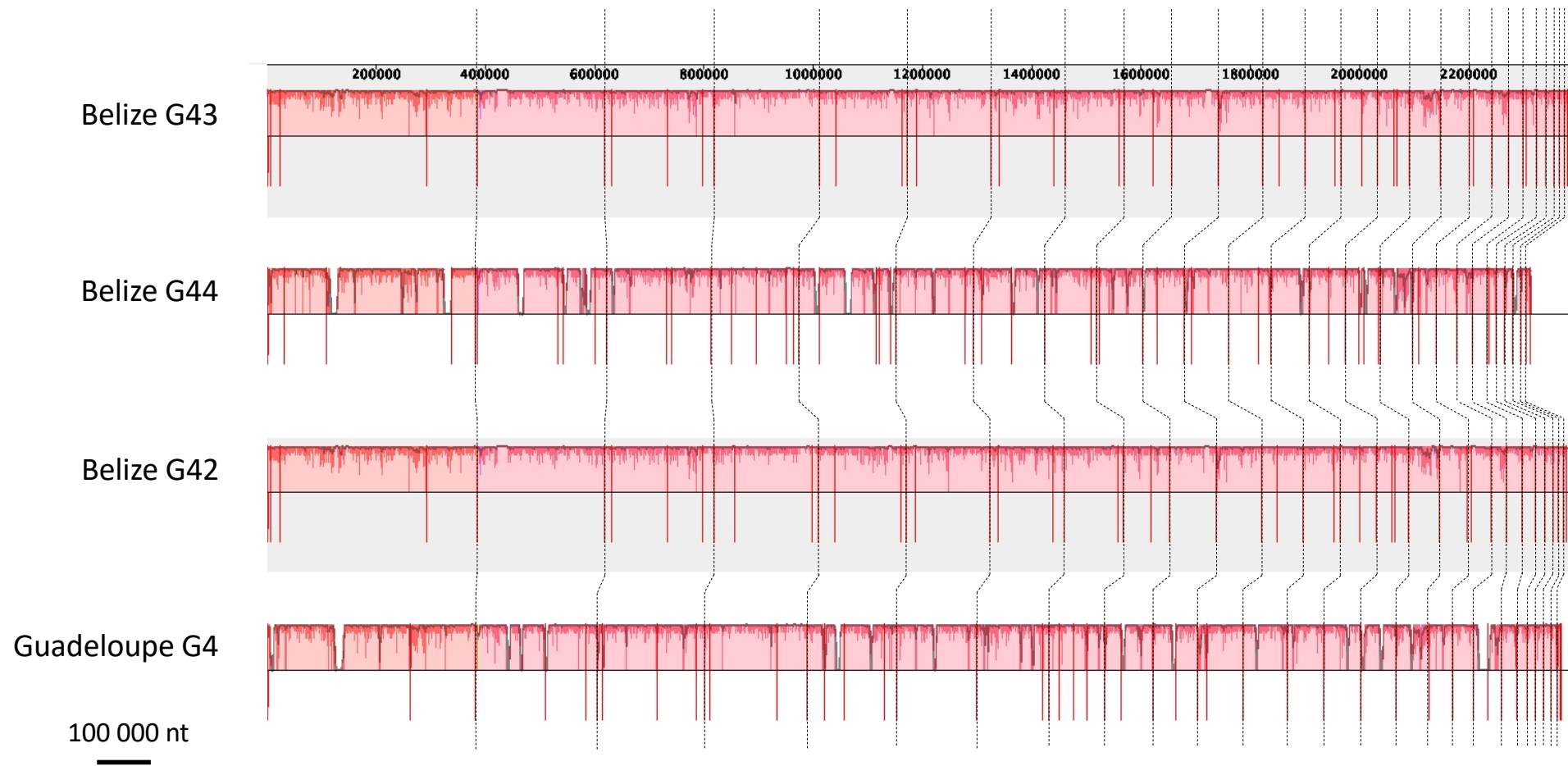

Supplement: Supplementary file 2 — Figure S2. [file MEN-24-0-s002.pdf]

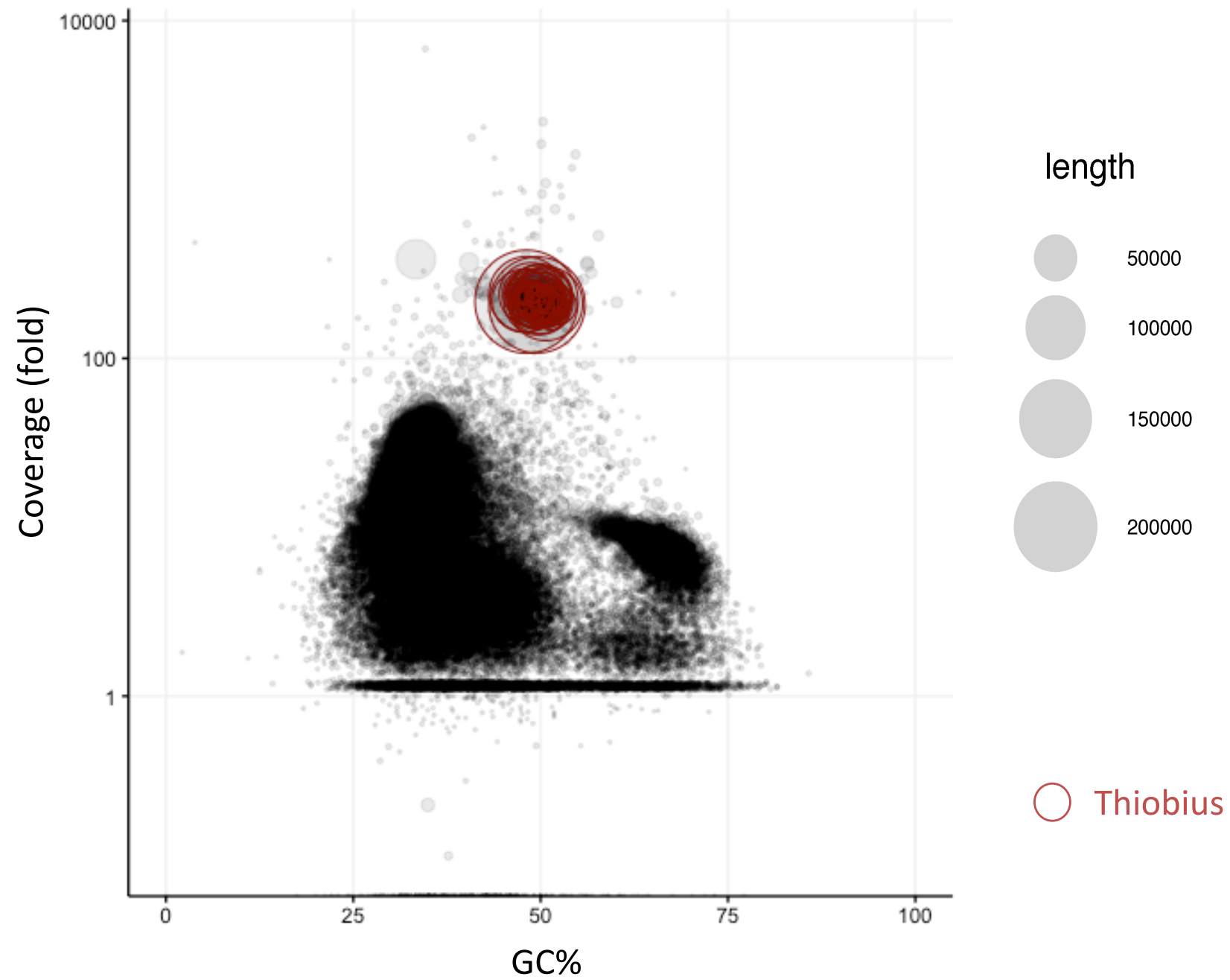

Supplement: Supplementary file 3 — Figure S3. [file MEN-24-0-s005.pdf]

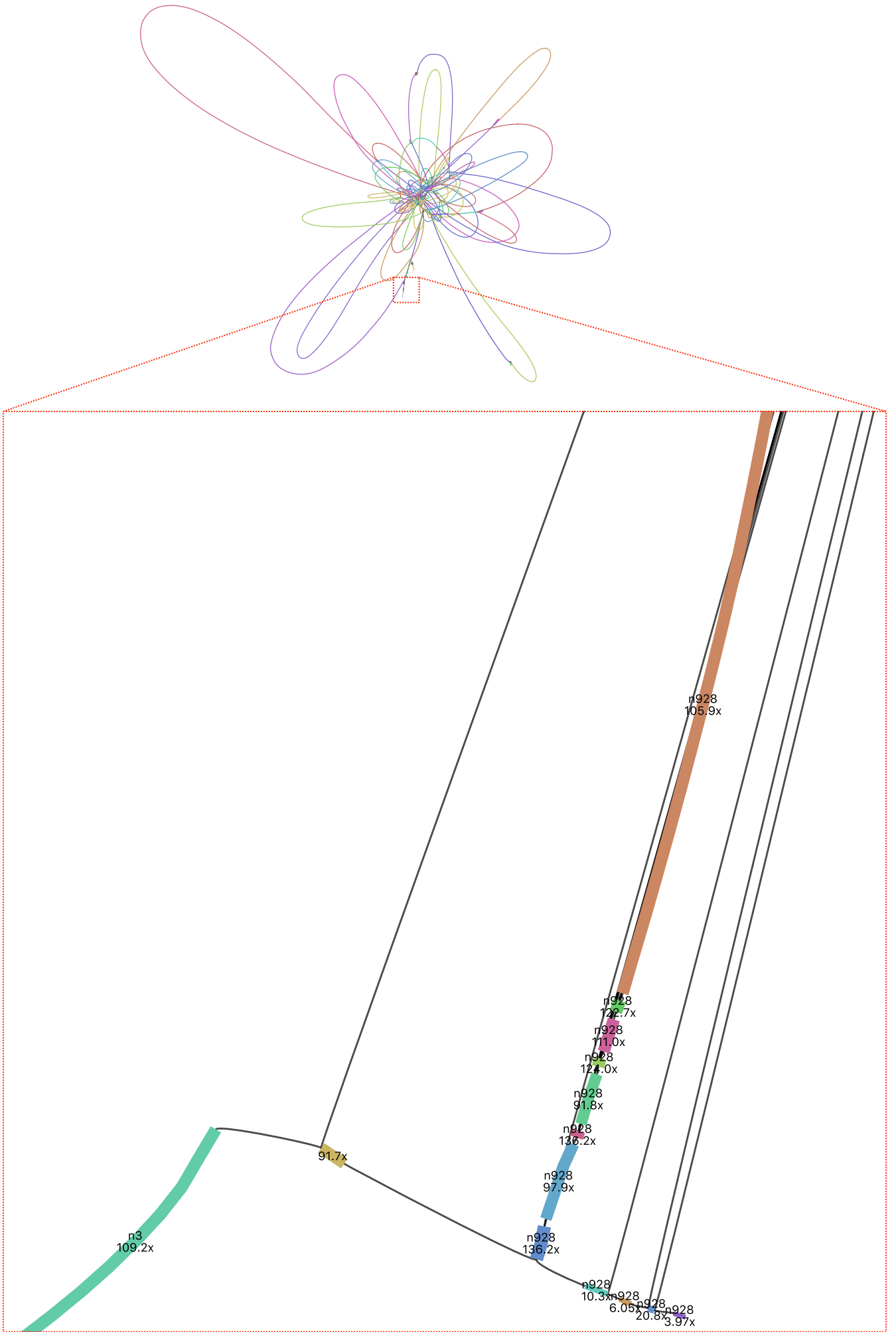

Supplement: Supplementary file 4 — Figure S4. [file MEN-24-0-s007.pdf]

Shared (%)

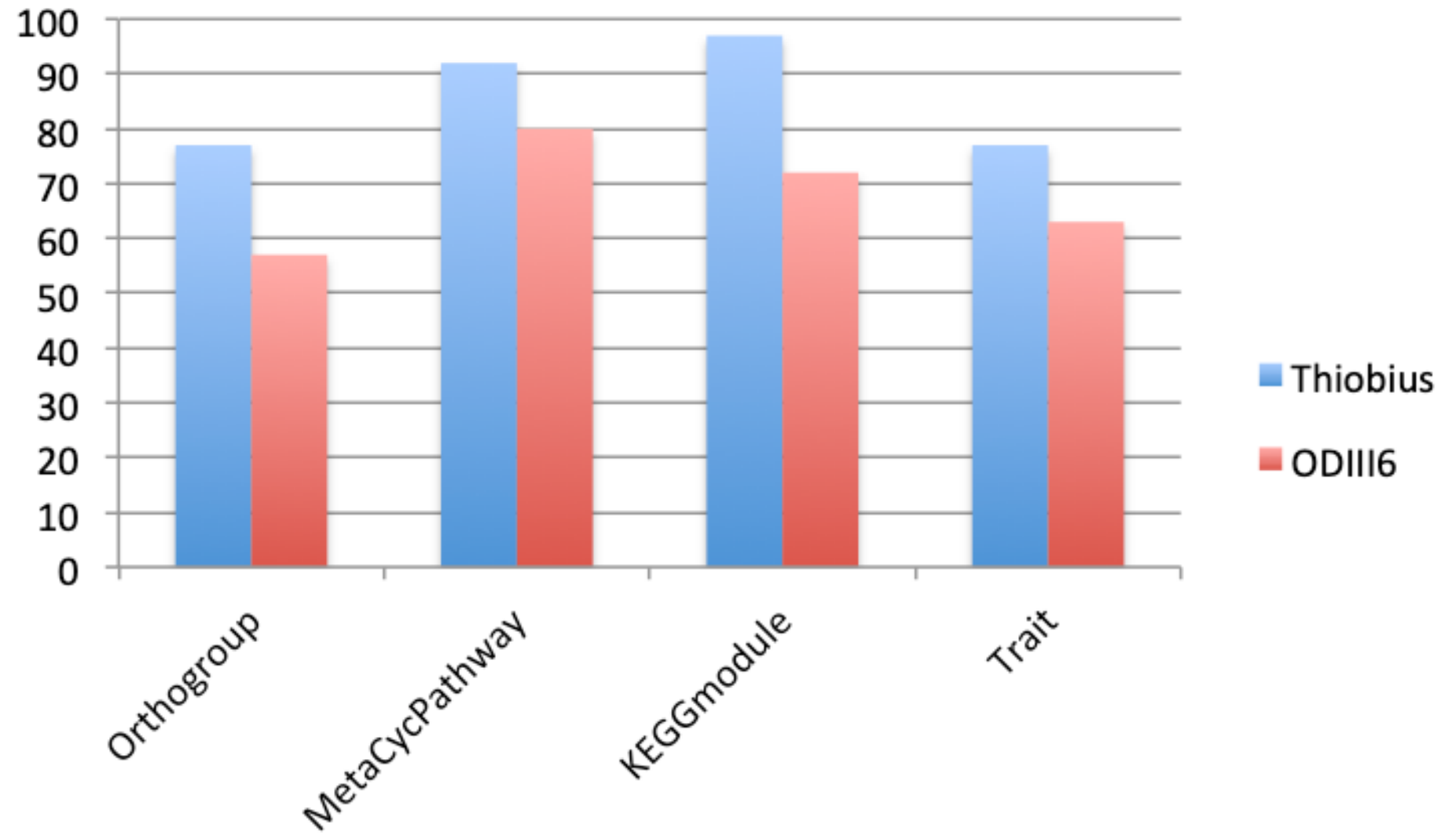

Supplement: Supplementary file 6 — Figure S6. [file MEN-24-0-s001.pdf]
