# Supplementary material for: Comparative genomics of a vertically transmitted thiotrophic bacterial ectosymbiont and its close free‐living relative
Source: Mol Ecol Resour. 2023 Nov 27;24(1):e13889. doi: 10.1111/1755-0998.13889 (PMC10952691; doi:10.1111/1755-0998.13889)

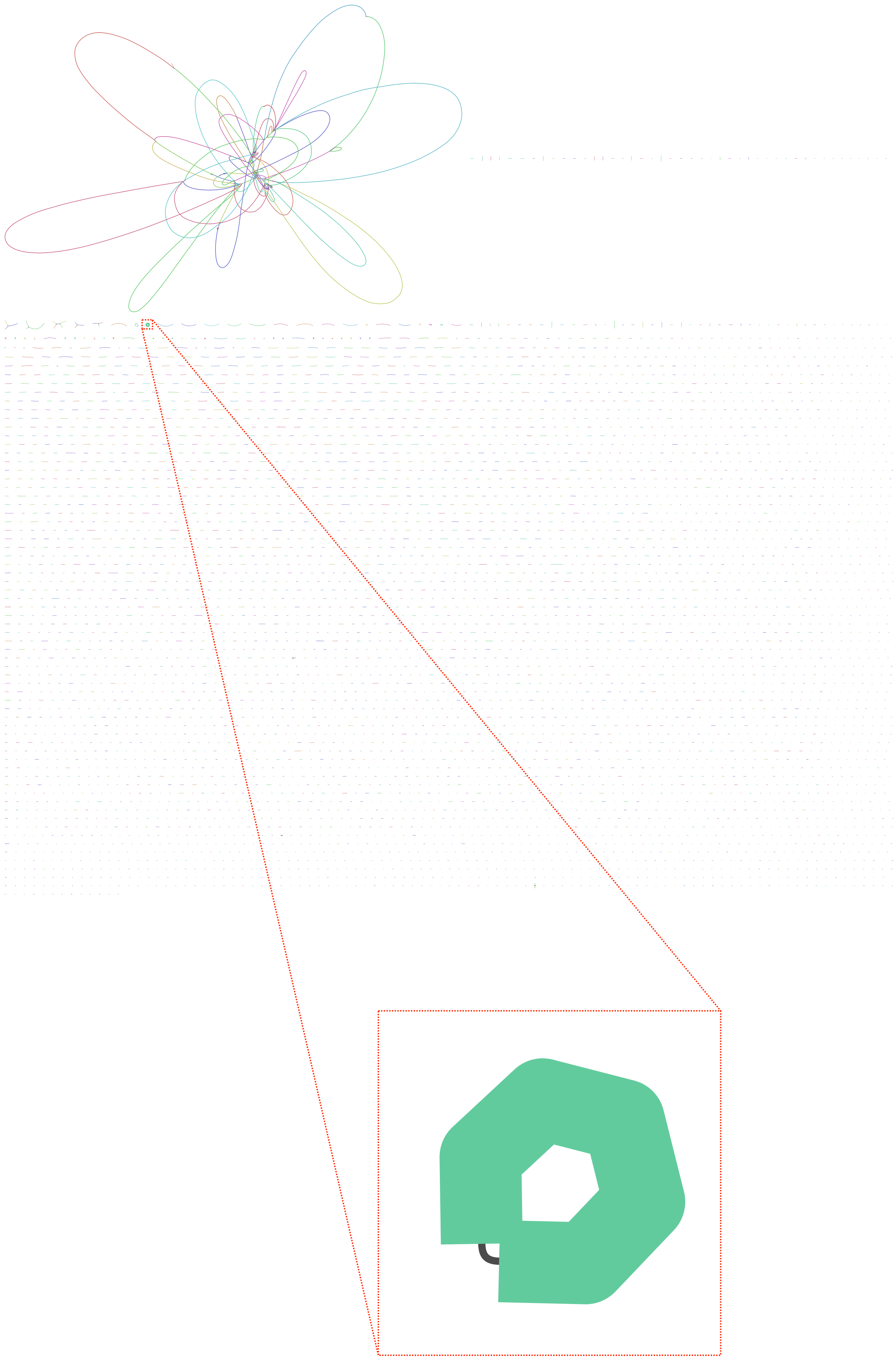

Supplement: Supplementary file 5 — Figure S5. [file MEN-24-0-s006.pdf]
